# The inflammasome in biomaterial‐driven immunomodulation

**DOI:** 10.1002/term.3361

**Published:** 2022-11-03

**Authors:** Daniela P. Vasconcelos, Artur P. Águas, Judite N. Barbosa

**Affiliations:** ^1^ i3S ‐ Instituto de Inovação e Investigação em Saúde Universidade do Porto Porto Portugal; ^2^ INEB ‐ Instituto de Engenharia Biomédica Porto Portugal; ^3^ ICBAS ‐ Instituto de Ciências Biomédicas Abel Salazar Universidade do Porto Porto Portugal; ^4^ UMIB ‐ Unit for Multidisciplinary Biomedical Research of ICBAS ‐ Instituto de Ciências Biomédicas de Abel Salazar Universidade do Porto Porto Portugal

**Keywords:** biomaterial, immunomodulation, inflammasome, inflammation, regenerative medicine

## Abstract

Inflammasomes are intracellular structures formed upon the assembly of several proteins that have a considerable size and are very important in innate immune responses being key players in host defense. They are assembled after the perception of pathogens or danger signals. The activation of the inflammasome pathway induces the production of high levels of the pro‐inflammatory cytokines Interleukin (IL)‐1β and IL‐18 through the caspase activation. The procedure for the implantation of a biomaterial causes tissue injury, and the injured cells will secrete danger signals recognized by the inflammasome. There is growing evidence that the inflammasome participates in a number of inflammatory processes, including pathogen clearance, chronic inflammation and tissue repair. Therefore, the control of the inflammasome activity is a promising target in the development of capable approaches to be applied in regenerative medicine. In this review, we revisit current knowledge of the inflammasome in the inflammatory response to biomaterials and point to the yet underexplored potential of the inflammasome in the context of immunomodulation.

## 1 THE INFLAMMASOME

In 2002 the research group of Jurg Tschopp, from the University of Lausanne, Switzerland, presented the concept of inflammasome for the first time. They found that the activation of the pro‐inflammatory protease, caspase‐1, requires a large molecular platform and named it the inflammasome (Martinon et al., 2002). The exciting report of this multiprotein complex that has the ability of sensing danger and thus initiate an inflammatory response led to a revival of the research on innate immunity (Dagenais et al., 2012).

It is now accepted that the stimulation of the innate immunity is not only due to the recognition of pathogen‐associated molecular patterns (PAMPs). It also requires the presence of danger‐associated molecular patterns (DAMPs) that are secreted by injured cells, and also, of lifestyle‐associated molecular patterns (LAMPs) that are the immunostimulatory molecular patterns in sterile inflammation associated, for example, with bioengineered implantable devices (Matzinger, 1994; Zindel & Kubes, 2020). Pattern‐recognition receptors recognize PAMPs, DAMPs and LAMPs, being the most studied the toll‐like receptors (TLRs). Activated TLRs will induce different signaling cascades resulting for instance in the production of inflammatory cytokines (Martinon et al., 2009).

**FIGURE 1:**
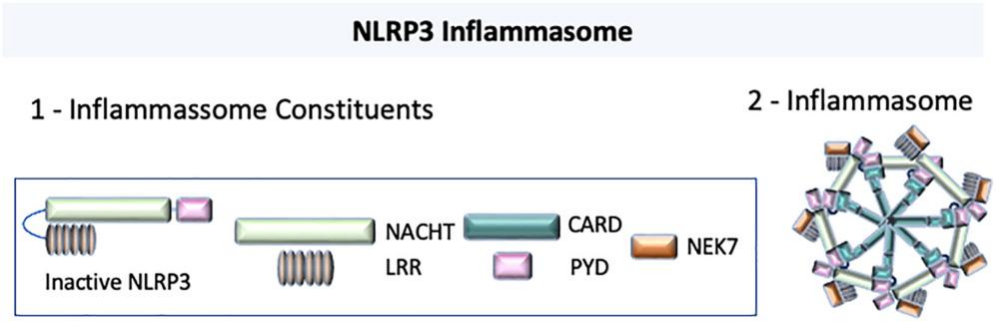
NLRP 3 Inflammasome activation. (1) Priming and activation stimuli induce NLRP3 oligomerization that recruits procaspase‐1 through apoptosis‐associated speck like protein containing a card (ASC). The inflammasome complex also recruits NIMA‐related kinase 7 (NEK7) that mediates interactions between adjacent NLRP3 subunits. (2) Within the inflammasome, procaspase‐1 undergoes autocatalytic processing, resulting in active caspase‐1. NLRP3, Nucleotide‐binding and oligomerization domain (NOD)‐, Leucine‐rich repeats (LRR) and pyrin domain‐containing protein 3 (PYD); NATCH, nucleotide‐binding domain; ASC, apoptosis‐associated speck‐like protein containing a caspase recruitment domain (CARD): NEK7, NIMA‐related kinase 7.

Inflammasomes can therefore be viewed as large cytoplasmic protein complexes (Figure 1) that have the ability of recognizing PAMPs, DAMPs and LAMPs. The inflammasome contains a nucleotide‐binding and oligomerization domain (NOD)‐like receptor (NLR), the adaptor molecule apoptosis‐associated speck like protein containing a card (ASC) and caspase‐1. The NLRs have the important function of surveying the intracellular microenvironment for the presence of metabolic perturbations, toxic substances and infection. When NLRs sense danger signals they undergo oligomerization and form macromolecules that have the ability of activating different inflammatory pathways (Zhong et al., 2013). The assembly of the NLRs, ASC and caspase‐1 induces the formation of a penta‐ or heptamer structure: the inflammassome (Strowig et al., 2012). The assembly of the inflammasome causes the activation of inflammatory caspases that will lead to the cleavage of pro‐interleukin (IL)‐1β and pro‐IL‐18 into IL‐1β and IL‐18, and also a type of cell death, pyroptosis (Lamkanfi & Dixit, 2014).

Different NLRs, upon activation, form inflammasomes as for example, NLRP1, NLRP3, NLRC4, NLRC5 and NAIP2 (Jha et al., 2017). The NLR protein 3 inflammasome (NLRP3 inflammasome), also recognized as cryopyrin or NALP3, is currently the better described inflammasome, being present predominantly in myeloid cells. The NRLP3 inflammasome is activated in response to stimulation by PAMPs, DAMPs and/or LAMPs of macrophages that will secrete extensive amounts of IL‐1β and IL‐18 through caspase‐1 activation (Latz, 2010; Ogura et al., 2006).

**FIGURE 2:**
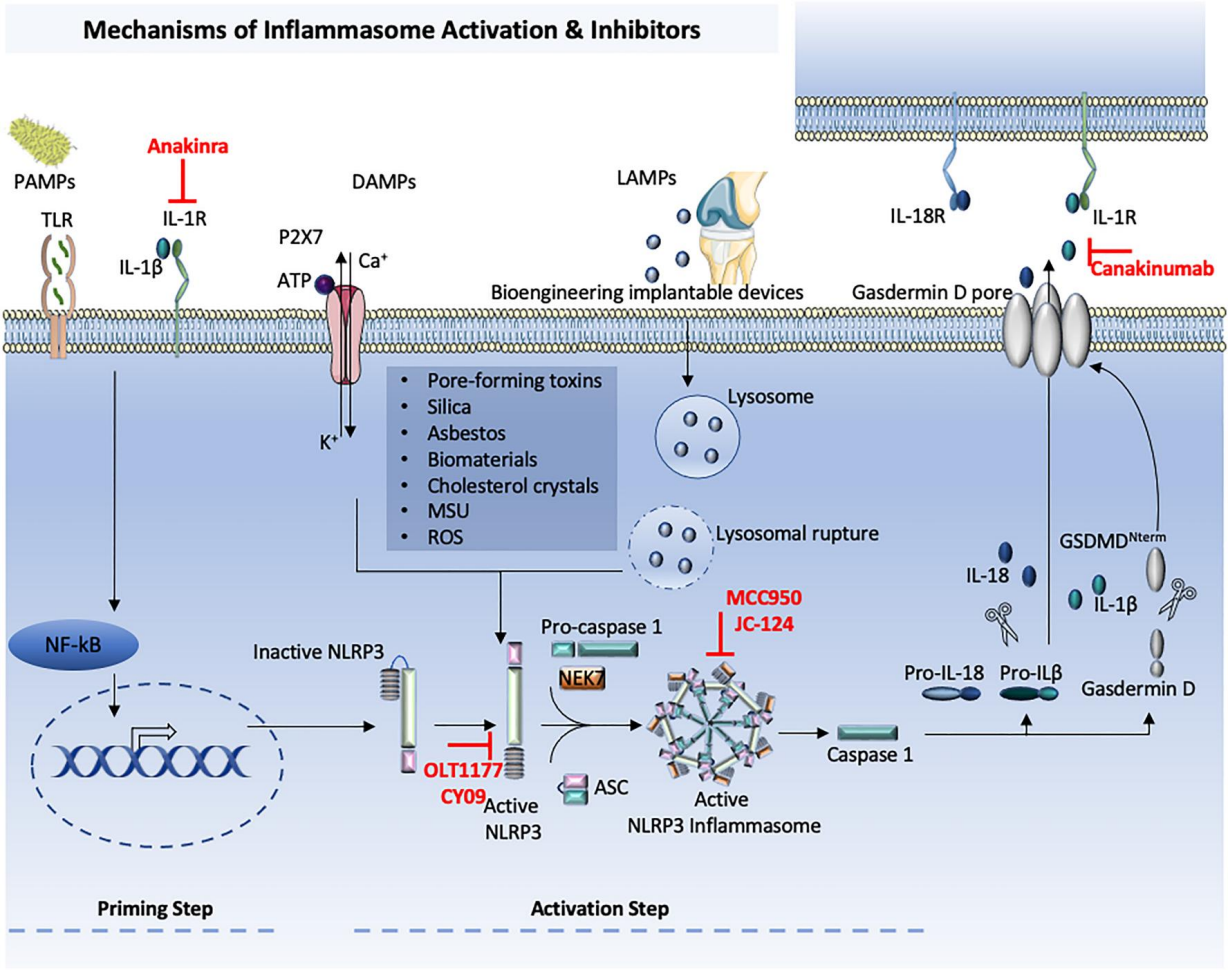
NLRP3 inflammasome activators and inhibitors. The signal 1 (priming; left) is provided by the activation of cytokines or pathogen‐associated molecular patterns (PAMPs), leading to the transcriptional upregulation of NLRP3 (Nucleotide‐binding and oligomerization domain (NOD)‐, Leucine‐rich repeats (LRR)—and pyrin domain‐containing protein 3 (PYD)) inflammasome components. Signal 2 (activation; right) is provided by numerous PAMPs, danger‐associated molecular patterns (DAMPs) (particulates, crystals, adenosine triphosphate (ATP)) and lifestyle‐associated molecular patterns (LAMPs) (Bioengineering implantable devices, cholesterol crystals) that activate multiple upstream signaling events. These include *K*+ efflux, Ca^2+^ flux, lysosomal disruption, reactive oxygen species (ROS) production. Oligomerization of the inflammasome activates caspase1, which in turn cleaves pro‐IL‐1b and pro‐IL‐18. Gasdermin D (GSDMD) is also cleaved and inserts its N‐terminal domain (GSDMD^Nterm^) into cellular membranes, forming pores to the release of the mature cytokines and inducing pyroptosis. ASC, apoptosis‐associated speck‐like protein containing a caspase recruitment domain; ATP, adenosine triphosphate; CNS, central nervous system; DAMPs, danger associated molecular patterns; IL‐, interleukin; LAMPs, lifestyle associated molecular patterns; LRR, leucine‐rich repeat; MSU, monosodium urate; NACHT, central nucleotide‐binding and oligomerization; NEK7, NIMA‐related kinase 7; NF‐k, nuclear factor‐kB; P2X_7_, P2X purinoceptor 7; PAMPs, pathogen associated molecular patterns; PYD, pyrin domain; ROS, reactive oxygen species; TLR, Toll‐like receptor.

The process of NLRP3 inflammasome assembly is not completely appreciated, but it is known that it is tightly regulated and its activation requires two‐step signals—first, it must be primed, and then activated (Figure 2) (Lamkanfi & Dixit, 2014). The first activation signal will cause de enhancement of the expression of the inflammasome constituents and will target proteins through the activation of the nuclear factor kappa light chain enhancer of activated B cells (Bauernfeind et al., 2009). The second activation signal will promote the oligomerization of the different components of the inflammasome, leading pro‐caspase‐1 to an autocatalytic processing causing caspase‐1 activation that will in turn cleave pro‐IL‐1β and pro‐IL‐18 into the mature and active forms. Caspase‐1 will also cleave gasdermin *D* that inserts its N‐terminal domain (GSDMD^Nterm^) into cellular membranes, permeabilizing the membrane to the release of the mature cytokines and to pyroptotic cell death (Evavold et al., 2018; Sborgi et al., 2016). The second NLRP3 activating signal comprises three main mechanisms: (i) formation of reactive oxygen species; (ii) lysosomal damage; and (iii) intracellular potassium efflux (Hafner‐Bratkovic & Pelegrin, 2018; Lima et al., 2013; Petrilli et al., 2007). NIMA‐related kinase 7 (NEK7), a serine‐threonine kinase, was recently considered to be crucial for NLRP3 inflammasome activation. Upon inflammasome activation, the NEK7‐NLRP3 synergy rises, and NEK7 oligomerizes with NLRP3 into a complex that is essential for ASC speck formation and caspase 1 activation. Thus, NEK7 seems to be an important component, in particular to the NLRP3 inflammasome (Schmid‐Burgk et al., 2016). Though most Pattern‐recognition receptors have limited specificity for one or few related PAMPs, DAMPs or LAMPs, NLRP3 is exclusive since it is triggered by a wide diversity of unrelated stimuli. NLRP3 is triggered both in pathogen infections and in sterile inflammation. Several different endogenous molecules that are indicative of injury or danger will stimulate the NLRP3 inflammasome, meaning that the NLRP3 inflammasome is able to detect sterile danger signals. These signals include extracellular adenosine triphosphate (ATP), uric acid and hyaluronan that are released by injured cells (Schroder & Tschopp, 2010). The expression of NLRP3 was detected in different cell types such as granulocytes, monocytes, macrophages, B and T lymphocytes, dendritic cells, osteoblasts and epithelial cells, suggesting an important role in the immune response against different threats. Therefore, the majority of studies on NLRP3 inflammasome have been performed in cells of the immune system (Lamkanfi & Kanneganti, 2010).

## 2 INFLAMMASOME DISORDERS AND ITS IMPLICATIONS IN HUMAN DISEASES

Inflammation is a protective response to a noxious stimulus. A defective inflammation leads to continuous infection, and excessive inflammation can originate disease. The inflammasome is one of the most relevant mediators in these processes (Davis et al., 2011). Consequently, inflammasome activity is highly controlled to avoid excessive cytokine production or cell death, being regulated at two levels, transcriptional (upregulation of inflammasomes components NLRP3, caspase 1 and pro‐IL‐1β) and post‐transcriptional (ubiquitylation, phosphorylation and sumoylation) (Yang et al., 2019). The expression of inflammasome sensors in resting cells is rather low and insufficient to be induced.

**FIGURE 3:**
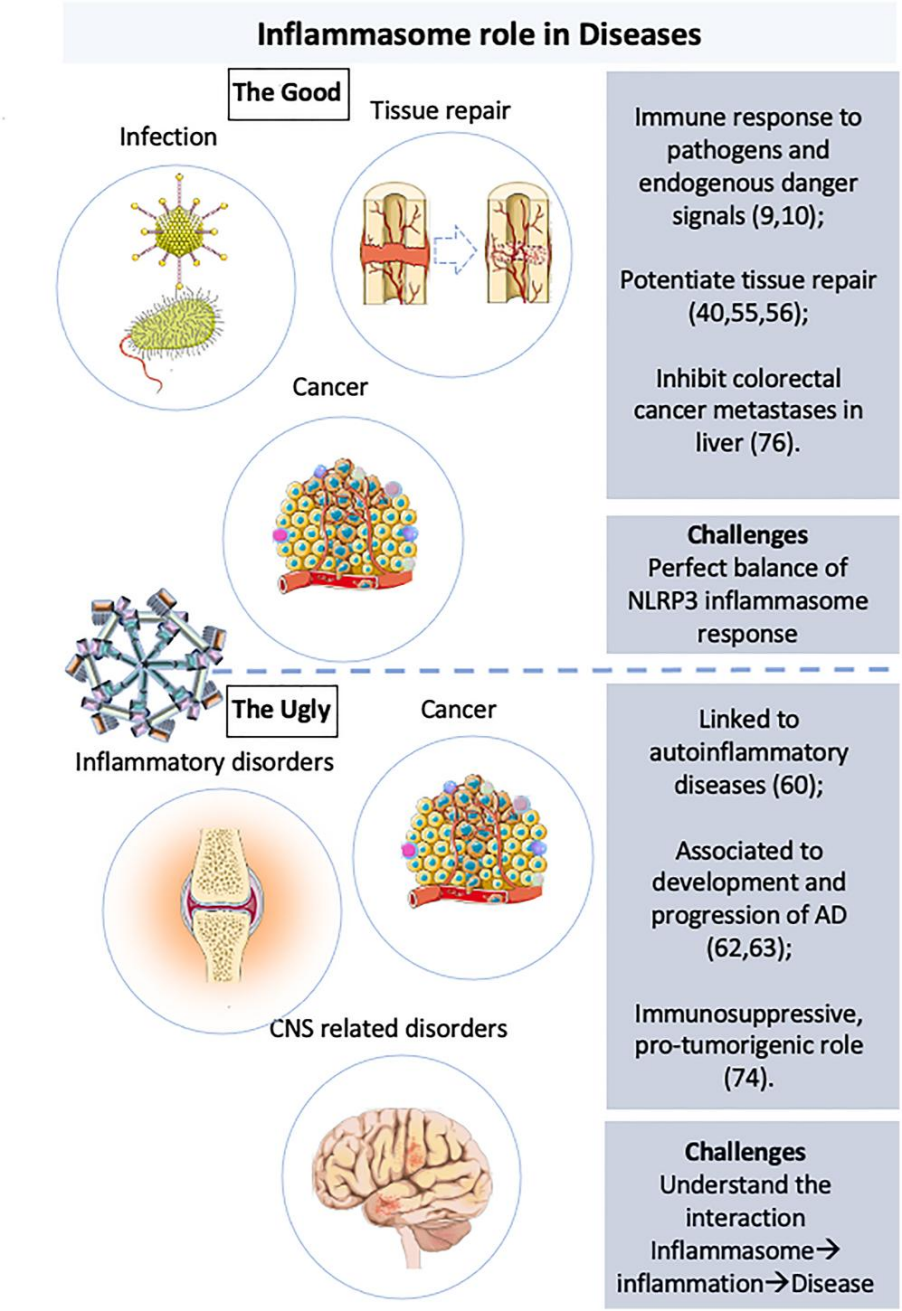
NLRP3 inflammasome role in disease. NLRP3 inflammasome has a key function in tissue repair and in host response not only to bacteria, fungi, viruses, and possible parasites, but also to danger‐associated molecular patterns (DAMPs) and lifestyle‐associated molecular patterns (LAMPs). Anomalous or exacerbated NLRP3 inflammasome activation is linked with the development of many diseases, such as, Alzheimer's Disease (AD), autoinflammatory diseases and cancer. The role of NLRP3 inflammasome in cancer is controversial, since there are evidences of a protective anti‐tumorigenic effect as well as a pro‐tumorigenic role depending of the cancer type. The correlation of NLRP3 inflammasome with a plethora of diseases hints a substantial interest in the scientific community to determine the effective NLRP3 inflammasome inhibitor

Inflammasome alterations have been associated to autoinflammatory, autoimmune and neurodegenerative diseases (multiple sclerosis, Alzheimer's disease (AD), Parkinson's disease), metabolic disorders (atherosclerosis, type 2 diabetes and obesity) and cancer, playing contributing roles in the beginning and development of those pathologies (Figure 3) (Strowig et al., 2012).

For instances, in AD is described an accumulation of amyloid‐β (Aβ) in plaques, aggregation of hyperphosphorylated tau in neurofibrillary tangles and neuroinflammation, leading to neurodegeneration (Ising & Heneka, 2018), the NLRP3 inflammasome has been related to the advance of Aβ pathology in AD models in mice. Furthermore, Venegas et al. (2017) showed that Aβ deposition is followed by innate immune system activation involving the formation of ASC specks in microglia in an inflammasome‐dependent manner. The use of an anti‐ASC antibody impaired the increase in Aβ pathology in an experimental model of AD. In addition, Yin et al. (2018) observed an increase of NLRP3 inflammasome in TgCRND8 mice, a mouse model of AD, inhibited by the treatment with JC‐124, a small molecule that harm NLRP3 oligomerization. More recently, Ising et al. (2019) demonstrated a decrease of NLRP3 inflammasome activity reduced tau hyperphosphorylation and aggregation in AD. The link between inflammasome activity and the pathophysiology of Aβ‐plaque and tau hyperphosphorylation suggests that the targeting of inflammasomes could represent a novel treatment for AD.

NLRP3 mutations have been linked with a rare hereditary autoinflammatory diseases known as cryopyrin‐associated periodic syndrome (CAPS). Cryopyrin‐associated periodic syndrome comprise three phenotypes: familial cold auto‐inflammatory syndrome, Musckle‐Wells syndrome, and chronic infantile neurological, cutaneous, and articular syndrome all of them, characterized by systemic inflammation (Hoffman & Wanderer, 2010). Local manifestations disturb multiple tissues such as skin, joints, muscles, eyes and the central nervous system. Cryopyrin‐associated periodic syndrome results from a gain‐of‐function mutation of the NLRP3 gene, leading to overproduction of IL‐1 (Dinarello, 2009). The rarity of CAPS and the similarity of symptoms with other diseases delay their accurate diagnosis. IL‐1 inhibitory agents, anakinra (a modified IL‐1RA that binds and antagonized the IL‐1 receptor), canakinumab (an IL‐1 neutralizing antibody) and rilonacept (ligand‐binding domains of the extracellular portions of IL‐1 receptor component) are the main therapeutic approach for CAPS. The blocking of IL‐1 leads to a rapid and continual reversal of daily symptoms, diminishing long‐term disease consequences (Hoffman, 2009; Lachmann et al., 2009; Sibley et al., 2012). The early diagnosis and treatment for CAPS patients are key to prevent organ damage.

Inflammation and immunosuppression are fundamental for cancer cells survival and progression. The role of NLRP3 inflammasome in cancer is arguable, since there are suggestions of a protective anti‐tumorigenic role but also a pro‐tumorigenic effect, depending of the cancer type (Hamarsheh & Zeiser, 2020; Kantono & Guo, 2017; Karki & Kanneganti, 2019). Kaplanov et al. (2019) demonstrated that IL‐1β in a 4T1 experimental mouse model of triple‐negative breast cancer has an immunosuppressive, pro‐tumorigenic role. Treatment with anti‐IL‐1β antibodies followed by anti‐PD‐1 antibodies fully abolished tumor development.

Chronic inflammation of mucosal surfaces and tissue injury observed in inflammatory bowel disease favor colorectal cancer. Huber et al. (2012) found that NLRP3 inflammasome detects tissue damage and promotes IL‐18 production that inhibits IL‐22 binding protein, leading to IL‐22 secretion and tumor growth. A possible explanation for these observations could be the fact that similar mediators and pathways in wound healing also have the ability to support tumorigenesis.

On the other hand, some authors demonstrated that NLRP3 inflammasome could also have an anti‐tumorigenic role: In divergence with the role of NLRP3 inflammasome in colorectal cancer cited above, Dupaul‐Chicoine et al. (2015) stated that IL‐18 release through NLRP3 pathway restrains colorectal cancer metastatic growth in liver by the increase of natural killer cell tumoricidal activity. The knowledge of the interaction between immunity and inflammation, inflammasomes, and cancer type may be crucial for the development of new therapies for cancer prevention and treatment.

The strong participation of the NLRP3 in inflammation and its roles in different kind of diseases makes it an attractive drug target (Figure 2). Current clinical approved treatment of NLRP3‐ related pathologies aims the inflammasome‐derived cytokine IL‐1β with IL‐1β antibodies or recombinant IL‐1β receptor antagonist: canakinumab and anakinra (Yang et al., 2019). However, they have disadvantages since inflammasome activation is important for host defense against a plethora of pathogens, and hence loss of IL‐1β can have deleterious effects on immune defense. Over the last years, some blockers of NLRP3 inflammasome pathway have been developed, few of which have been validate in animal models. MCC950 is a compound that specifically inhibits NLRP3 inflammasome activation, but its molecular mechanism has not been fully elucidated (Coll et al., 2015). Cy‐09 and OLT1177 can inhibit the ATPase activity of the NACHT domain, which is critical for NLRP3 oligomerization (Jiang et al., 2017; Kuwar et al., 2019). JC‐124 blocks the expression of NLRP3, ASC, Caspase 1 and pro‐IL‐1b, but its molecular mechanism are still under investigation (Marchetti et al., 2015). Due the number of individuals with serious conditions driven by NLRP3, there is a strong motivation for the discovery and clinical development of molecules that selectively antagonize NLRP3. Although these inhibitors have shown therapeutic potential, the food and drug administration and other regulatory agents did not approve any of them. In addition, the NLRP3 structure and activation mechanisms are still poorly understood which has delayed the development of novel therapeutics. Nevertheless, there are pre‐clinical evidences that the pharmacological inhibition of the NLRP3 inflammasome pathway has for example, a neuroprotective role in different disease models, therefore providing convincing arguments to further evaluate the potential of targeting the NLRP3 inflammasome (Jose et al., 2022).

## 3 THE ACTIVATION OF THE INFLAMMASOME BY BIOMATERIALS

Tissue engineering and regenerative medicine are focused in the development of therapies to regenerate or replace injured, diseased, or defective cells, tissues, or organs to restore or establish function and structure (Daar & Greenwood, 2007). Many of the developed approaches are biomaterial‐based which makes the understanding of innate and adaptive immune responses critical for the success of their application (Sefton et al., 2008). Great advances in this area have been achieved by James Anderson that has presented us a pathologist perspective on the foreign body reaction (FBR) to biomaterials (Table 1) in several manuscripts that are still today a reference for researchers in this field (Anderson, 1988, 2001; Anderson et al., 2008). More recently, Franz et al. (2011) presented a thorough review on the immune responses to biomaterials and were one of the first authors to address the importance of developing biomaterials capable of modulating the immune response and to present the concept of immunomodulatory biomaterials, however the concept of inflammasome activation by biomaterials was not herein explored. A few years latter Christo et al. (2015) present a detailed review on innate immunity and biomaterials and discuss the role of the inflammasome in the inflammatory response to biomaterials.

**TABLE 1:** The foreign body reaction (FBR) to biomaterials (Anderson, 2001; Anderson et al., 2008; Christo et al., 2015; Franz et al., 2011)

Phases of the foreign body reaction	Brief description	Timescale
Biomaterial implantation	The implantation of a biomaterial or biomedical device causes injury in tissues or organs leading to the onset of an inflammatory response.	*t* = 0
Protein Adsorption	Blood proteins will adsorb to the surface of the biomaterial leading to the activation of the coagulation and complement system and to the activation of platelets.	*t* > 1 s
Inflammatory cells recruitment	Inflammatory cells, initially predominantly polymorphonuclear leukocytes (PMNs) are recruited to the implant site. Activated PMNs secrete chemokines that act as chemoattractants to monocytes, macrophages, immature dendritic cells and lymphocytes.	*t* = 60 min
Cell adhesion to the biomaterial	Monocytes differentiate into macrophages that will adhere to the surface of the biomaterial and secrete reactive species in an attempt to degrade and phagocyte the material. In larger materials, macrophages fuse and form foreign body giant cells (FBGCs).	*t* = 1–15 days
Fibrous Capsule formation	Macrophages and FBGCs release factors such as transforming growth factor beta (TGF‐b) and platelet‐derived growth factor (PDGF) that recruits and activates fibroblasts and endothelial cells. Activated fibroblasts will synthesize collagen leading to the formation of a fibrous capsule and consequently biomaterial encapsulation.	*t* = 3–4 weeks

The process of implantation of a biomaterial causes injury to tissues that will release DAMPs and may lead to the activation of the inflammasome pathway. Tissue resident macrophages will be one of the first immune cells to respond to injury. When activated by DAMPs, these tissue resident macrophages will release chemokines and cytokines that will prime the recruitment of polimorphonuclear leukocytes and monocytes to the injured site further activating the inflammatory response (Raziyeva et al., 2021). In Table 2 we summarize some general concepts of the inflammatory response related to the host response to biomaterials.

**TABLE 2:** General concepts of an inflammatory response related to the host response to biomaterials (Coll et al., 2015; Elmore, 2007; Land, 2020; Williams, 2017; Zotova et al., 2016)

General concepts of an inflammatory response	
Acute inflammation	Initial phase of the inflammatory response of relatively short duration (minutes to days). Mainly characterized by the exudation of plasma proteins and fluid and by the emigration of leukocytes, mainly polymorphonuclear leukocytes (PMNs), from blood vessels to the injured site.
Chronic inflammation	Occurs when the inflammatory stimuli persists. Monocytes are recruited to the inflammatory environment and differentiate into macrophages. Macrophages become the predominant inflammatory cell. The constant release of inflammatory mediators leads to permanent activation of macrophages, and the production of chemokines leads to the recruitment of additional inflammatory cells.
Systemic inflammation	Generalized inflammatory response throughout the whole body. Characterized by the inflammatory reactivity of endotheliocytes, plasma and blood cell factors.
Sterile inflammation	Sterile inflammation is an inflammatory response that occurs in the absence of microorganisms. Is associated with the recognition of molecules released from injured cells (DAMPs: Damage‐associated molecular patterns). Biomaterials induce sterile inflammation.
PAMPs	Pathogen‐associated molecular patterns: Molecular structures produced by microorganisms that are recognized as foreign by the innate immune system.
DAMPs	Damage‐associated molecular patterns: Molecules released upon non‐physiological cell death, damage, or stress that are indicative of danger and are sensed by the innate immune system and activate immune cells. There are also exogenous DAMPs such as airborne particles.
PRRs	Pattern recognition receptors: Expressed on leukocytes interact with PAMPs and DAMPs leading to leukocyte activation. There are different families of PRRs such as toll‐like receptors (TLRs) and NOD‐like receptors (NLRs).
Apoptosis	Apoptosis is a process of programmed cell death. This process occurs normally during development and aging but it has also an important role in immune responses.
Pyroptosis	Pyroptosis is an inflammatory type of programmed cell death that is typically elicited by the inflammasome. It is characterized by the permeation of the plasma membrane leading to the subsequent release of intracellular contents.

Inflammasome stimulation by biomaterials is being investigated mainly with bioengineered nanomaterials (Christo et al., 2016; Silva et al., 2017). It has been reported as a component of the inflammatory response to several biomaterials such as gold nanoshells (Nguyen et al., 2012), silver nanoparticles (Yang et al., 2012) and chitin/chitosan (Bueter et al., 2011) but these studies are based mainly in the assessment of the production of IL‐1β. There are however more detailed studies available in the literature that address the biological effects of different biomaterials, mainly nanomaterials, on immune cells and on the NLRP3 inflammasome activation that we summarize in the following paragraphs and review on Table 3. On the following section of this manuscript we explore some applications on the targeting of the inflammasome pathway in the context of tissue engineering and regenerative medicine.

**TABLE 3:** Examples of the effect of biomaterials in the inflammasome pathway activation

Biomaterial	Inflammasome Response	Reference
Nano‐scale based biomaterials		
Carbon nanoparticles	Activation of caspase‐1 increased IL‐1β production	Reisetter et al. (Reisetter et al., 2011)
Amino‐functionalized polystyrene nanoparticles	Assembly of the NLRP3 inflammassome increased IL‐1β production	Lunov et al. (Lunov et al., 2011)
Carboxyl‐ functionalized or non‐functionalized polystyrene nanoparticles	No effect on the inflammmasome pathways was observed	
Silica nanoparticles	Increased levels of IL‐1β through inflammasome pathway activation	Gómes et al. (Gomez et al., 2017)
Titanium dioxide nanoparticles	Increased gene expression of NLRP3, caspase‐1 and IL‐1β	Abbasi‐Oshaghi et al. (Abbasi‐Oshaghi et al., 2019)
Chitosan‐aluminum nanoparticles	Activation of the NLRP3 inflammassome increased IL‐1β production	Lebre et al. (Lebre et al., 2018)
Micro‐scale based biomaterials		
Cobalt‐Chromium‐Molybdenum alloy microparticles	Irregular and larger microparticles induced higher levels of IL‐1β through inflammasome pathway activation.	Caicedo et al. (Caicedo et al., 2013)
Hydroxyapatite microparticles	Smaller and needle‐shaped microparticles lead to activation of the NLRP3 inflammassome increased IL‐1β production	Lebre et al. (Lebre et al., 2017)
Microspheres of poly(methyl methacrylate)	Activation of caspase‐1 further secretion of IL‐1β	Malik et al. (Malik et al., 2011)
Large‐scale based biomaterials		
Collagen 3D scaffolds	Induced assembly of the NLRP3 inflammasome increased IL‐1β secretion	Court et al. (Court et al., 2019)
3D chitosan scaffolds	Impaired NLRP3 inflammasome assembly	Vasconcelos et al. (Vasconcelos et al., 2019)

Based on the available literature it can be concluded that there is a clear tendency for the activation of the inflammasome pathway by nano‐ and micro‐particles. Reisetter et al. (2011) have performed in vitro studies with macrophages exposed to carbon black nanoparticles that triggered inflammasome activation evaluated by the activation of caspase‐1 and ensuing IL‐1β production. Lunov et al. (2011) have demonstrated in vitro that amino‐functionalized polystyrene nanoparticles caused the assembly of the NLRP3 inflammassome leading to the production of IL‐1β. However, this activation was not observed for carboxyl‐ or non‐functionalized particles. Gomez et al. (2017) described that silica nanoparticles induced the release of pro‐inflammatory cytokines with the involvement of NLRP3 inflammasome constituints. Caicedo et al. (2013) investigated the importance of shape and size of biomaterial particles on inflammasome activation using Cobalt‐Chromium‐Molybdenum alloy and concluded that the most irregular and the larger particles induced the highest levels of IL‐1β release through inflammasome pathway activation. Abbasi‐Oshaghi et al. (2019) investigated the impact of titanium dioxide nanoparticles (TiO_2_NPs) on the intestine and liver of normal animals and observed that TiO_2_NP had the ability to trigger the NLRP3 inflammasome pathway both in the intestine and liver. Their studies on NLRP3 inflammasome gene expression revealed that NLRP3, caspase‐1 and IL‐1β were significantly increased in the different experimental groups (10, 50, and 100 mg/kg) when compared to values of control animals. Baron et al. (2015) demonstrated that nano‐sized inorganic metal oxides such as silica dioxide (SiO_2_) and titanium dioxide (TiO_2_) were able to activate in macrophages the NLRP3 inflammasome. Both SiO_2_ and TiO_2_ nanoparticles promoted the release of mature IL‐1β by macrophages via the active secretion of ATP to the extracellular space. Lebre et al. (2017) assessed the impact of size and shape of hydroxyapatite (HA) particles in the innate immune response, and concluded that shape and size influence the assembly of the NLRP3 inflammasome and IL‐1β secretion, being the needle‐shaped and smaller HA particles the ones that increased cytokine secretion, whereas larger particles did not. These authors, in a different experimental study, have also concluded that chitosan‐aluminum nanoparticles (CH‐Al NPs) formulations lead to NLRP3 inflammasome activation, augmenting IL‐1β release (Lebre et al., 2018). Malik et al. (2011) have demonstrated that the inflammasome in involved in the advance of the FBR. Their studies explored the role of the different inflammasome constituents to biomaterials response, irrespective of size, and showed that inflammasome assembly can be triggered by physical membrane contact. They have used NLRP3, ASC, NLRC4 and caspase‐1 deficient mice and concluded that microspheres of poly(methyl methacrylate) can activate the NLRP3 inflammasome causing the formation of an inflammatory exudate dependent on the inflammasome constituents NLRP3, ASC and caspase‐1, leading to the activation of caspase‐1 and further secretion of IL‐1β. Mukherjee et al. (2018) reported that graphene oxide with small or large lateral dimensions elicits caspase‐dependent IL‐1β expression, indicative of inflammasome activation. Christo et al. (2016) have developed model surfaces with precise nanotopography and surface chemistry. They have used macrophages from genetically engineered mice deficient in the inflammasome constituints ASC, NLRP3 and AIM2. Their results suggested that the inflammasome components ASC, NLRP3 and AIM2 have a role in regulation of the activation and adhesion of macrophage in reaction to surface nanotopography and chemistry. Turley et al. (2021) produced several solutions of chitosan polymers with different percentages of deacetylation and concluded that higher deacetylation leads to an increased NLRP3 inflammasome activation.

Although significantly fewer, studies using biomaterials too large to be phagocytosed were also performed to evaluate the role of the inflammasome in phagocytosis‐independent inflammatory response to biomaterials. Court et al. (2019) documented that collagen 3D scaffolds induced assembly of the NLRP3 inflammasome causing an increased IL‐1β secretion observed in human macrophages. We have used 3D chitosan scaffolds (Vasconcelos et al., 2019) and observed that these scaffolds impair NLRP3 inflammasome assembly in macrophages. Remarkably, the results that we obtained are different of other studies reported in the literature that demonstrate that chitosan is an inducer of the NLRP3 inflammasome in nanoscale chitosan products. Taken together, these results suggest that phagocytosis could be important in inflammasome assembly and activation, and suggest a different role of the inflammasome in the host response to nano‐ and large‐scale biomaterials.

Interestingly, the macrophage response to biomaterials is size‐dependent. Macrophages have the ability to phagocyte small fragments and particles (<10 μm). When the particle size is larger (between 10—100 μm) macrophages fuse and form foreign body giant cells (FBGCs) that will engulf and digest the particle. For even larger particles, the digestion occurs via extracellular degradation in a phenomenon described as frustrated phagocytosis (Sheikh et al., 2015). This concept was first described by Henson et al. (Henson, 1971) and has great importance in the host response to biomaterials. It has been demonstrated that FBGCs adherent at the surface of implanted biomaterials present a reduced phagocytic activity but an enhanced degradative capacity through the increased secretion of reactive species (Franz et al., 2011). As far as our knowledge, there are no studies that relate the formation of FBGCs, frustrated phagocytosis and the inflammasome activation which in our opinion could be a breakthrough in this area of research.

In conclusion, there is not yet a clear understanding on how the inflammasome pathway will be triggered by different biomaterials, despite recognizing its key importance. More detailed studies, namely on the importance of the different inflammasome constituents, are required to fully appreciate the importance of the activation of this pathway in the inflammatory response to biomaterials. The modulation of inflammasome activity appears nonetheless as an important strategy to develop effective approaches for successful biomaterial integration that is a central challenge in regenerative medicine research.

## 4 THE POTENTIAL OF THE INFLAMMASOME IN IMMUNOMODULATION

There is growing indication that the immune response has an important influence in the process of tissue repair and regeneration (Eming et al., 2009). Activation of the inflammasome can either lead to the resolution of the inflammatory response and healing, or can be continuous, leading to chronic disease or fibrosis (Artlett, 2013). Therefore, inflammasomes are considered important regulators of the intensity of the inflammatory response, as well as regulators of the ensuing tissue repair (Ouyang et al., 2013).

As described in the beginning of this manuscript, the assembly and activation of the NRLP3 inflammasome leads to the secretion of robust amounts of IL‐1β and IL‐18 via caspase‐1 activation. Interestingly, caspase‐1 activated cells secretome contains important proteins to restore tissue homeostasis, such as fibroblast growth factor (FGF)‐2 that has an important role in tissue repair and homeostasis, therefore directly associating inflammation to regeneration (Baroja‐Mazo et al., 2014; Keller et al., 2008). Additionally, both IL‐1β and IL‐18 act on several innate and adaptive immune cells and regulate their activation, differentiation and migration to local tissues. Non‐hematopoietic cells as for example, endothelial and epithelial cells are also targeted to contribute to tissue repair (Palomo et al., 2015). Therefore, understanding the signaling that is elicited by inflammasomes can be used to advance healing, making the inflammasome a rather promising target in the research area of immunomodulatory biomaterials directed tissue repair. An improved understanding of the inflammasome biology can potentially contribute to key pathways that regulate immunity, inflammation and homeostasis. Therefore, effective control of the inflammasome activity has become a potential therapeutic strategy and may become a milestone in bioengineering.

Although research on the targeting of the inflammasome and/or inflammasome constituents is still in the beginning, there are some studies reported in the literature that support the potential of this approach and that are revealing some interesting outcomes in different areas of research that include skin wound healing, bone healing, liver regeneration, pancreatic island transplantation and also in neuronal injuries.

Weinheimer‐Haus et al. (2015) showed that NLRP3 and caspase‐1 null mice submitted to a cutaneous wound excision presented a delay in re‐epithelization, granulation tissue formation and angiogenesis, demonstrating the importance of NLRP3 signaling in the early events of wound healing. Ito et al. (2018) further documented that NLRP3 signaling is important for the skin healing process. They observed that NLRP3 and ASC knockout (KO) mice presented an impaired wound repair when comparing to the wild type (WT) animals. They further concluded that the administration of ATP, which is a ligand for NLRP3, leads to the activation of inflammasomes causing the up‐regulation of the expression of pro‐inflammatory cytokines and accelerates the wound healing. Therefore, identifying molecules that have the ability to modulate the inflammasome assembly and activation, or inflammasome‐mediated products, can reduce fibrosis and promote wound healing (Artlett, 2013). Chen et al. (2022) investigated the early inflammatory response to titanium screws in bone, and concluded that the administration of ipriflavone, a natural isoflavone, decreased the activation of the NLRP3 inflammasome in macrophages. They reported the interesting osteoimmunomodulatory effect of ipriflavone that targets the NLRP3 inflammasome, thus having an anti‐inflammatory effect. Xu et al. (2020) developed a polyelectrolyte complex nanoparticle using chitosan and carboxymethyl chitosan to be used as a doxycycline carrier for periodontal disease. They observed that the developed nanoparticles down‐regulated both gene and protein levels of NLRP3 inflammasome together with a decrease of IL‐1β levels. Sartoretto et al. (2019) using an in vivo mouse model of noncritical bone defects in tibias, using WT mice and ASC KO mice, showed that bone de novo deposition is affected by ASC‐dependent inflammasome signaling, since ASC KO animals presented a delayed healing and were not able to completely heal the tibia defects. Ando et al. (2017) demonstrated that liver regeneration is impaired by deficiency of NLRP3 signaling. In NALP3 KO mice the liver‐to‐body weight ratio was considerably reduced when compared to the WT after partial hepatectomy. Moreover, the levels of IL‐1β, TNF‐α, and IL‐6 decreased in the liver of NALP3‐KO mice when compared to WT mice. These results point out that NALP3 signaling is necessary for the initiation of an inflammatory response and for the improvement of liver regeneration. Moreover, treating WT mice with ATP augmented the liver‐to‐body weight ratio. Matsuoka et al. (2020) investigated the use of MCC950 to inhibit the NLRP3 inflammasome in a rodent model of pancreatic islet transplantation and concluded that the survival and viability of transplanted islets is improved after NLRP3 inhibition. Mousavi et al. (2019) in a rat model of spinal cord injury used cell therapy with Schwann cells with the aim of targeting NLRP inflammasomes. They concluded that the reduction of the activation of the inflammasome caused by the cell therapy had a neuroprotective effect. Barone et al. (2022) combining a NLRP3 inhibitor (MCC950) with an implantable electronic medical devices, demonstrated the prevention of FBR without affecting tissue repair, in an in vivo nerve injury model. The authors showed that local inhibition of NLRP3 inhibits FBR, allowing full healing of the tissue, in comparison with the conventional treatments (dexamethasone), which also blocks FBR but at the same time impair tissue repair.

Although so far scarce, reports on the importance of the inflammasome in regenerative processes showed encouraging results, indicating the need to pursue this promising area of research. The combination of inflammasome modulators with biomaterials will most likely yield encouraging results for regenerative medicine applications.

## 5 CONCLUSIONS

The implantation of a biomaterial causes tissue damage. Damaged cells will release the so‐called “danger signals” that can be identified by the inflammasome. Therefore, implanted biomaterials are potential activators of the inflammasome pathway. The role of the inflammasome in the host response to biomaterials as well as the stimulation of inflammasomes by implanted biomaterials needs a more in‐depth investigation since it is still poorly understood.

It is important to clarify several different aspects on the biomaterial—inflammasome interactions, such as for example,:

(i) What are the differences between nano‐ or micro‐particles and large‐scale biomaterials, and is the process of phagocytosis essential in the process of inflammasome assembly and activation?
(ii) How can biomaterial surface characteristics modulate innate immune signaling pathways?
(iii) How biomaterial‐adherent cells affect these signaling pathways and is surface chemistry a key aspect?
(iv) How does the inflammasome activation influences the progression of the foreign body response to biomaterials?
(v) What is the importance of the different inflammasome constituents?
(vi) What can we learn using genetically modified mice deficient in these key signaling pathways?

It is in our opinion important to better understand how to control the host response by targeting specific points in these innate immune signaling pathways to improve biomaterials biocompatibility Therefore, further research into how biomaterials influence the inflammasome assembly and activation are of great interest.

It is now commonly recognized that the immune system is of major importance in the coordination of an adequate repair process, and since inflammasomes participate in the innate immune response, it is predictable that they have an important role in tissue repair/regeneration. The assembly of the inflammasome will not only lead to the production of pro‐inflammatory cytokines but also to the regulation of the extracellular levels of proteins involved in the processes of tissue repair and cytoprotection.

A thorough understanding of the inflammasome biology will give us key information on different pathways that regulate immunity, inflammation and homeostasis. It is our belief that a successful modulation of the activity of the inflammasome will become a milestone in bioengineering, and namely in the field of tissue repair and regeneration. There is a growing interest in finding effective approaches that will be able to selectively inhibit the inflammasome pathway.

## AUTHOR CONTRIBUTIONS

**Daniela P. Vasconcelos**: Writing ‐ Original draft. **Artur P. Águas**: Writing ‐ Review & Editing. **Judite N. Barbosa**: Writing ‐ Original draft; Review & Editing.

## CONFLICT OF INTEREST

The authors have no conflict of interest to declare.

## Data Availability

Data sharing not applicable to this article as no datasets were generated or analyzed during the current study.
